# Prediction of the Released Mechanical Energy of Loaded Lap Shear Joints by Acoustic Emission Measurements

**DOI:** 10.3390/s24227230

**Published:** 2024-11-12

**Authors:** Thomas Wolfsgruber, Martin Schagerl, Christoph Kralovec

**Affiliations:** Institute of Structural Lightweight Design, Johannes Kepler University Linz, 4040 Linz, Austria; martin.schagerl@jku.at (M.S.); christoph.kralovec-roedhammer@jku.at (C.K.)

**Keywords:** structural health monitoring, acoustic emission, damage evaluation, hybrid lap shear joint, released energy prediction

## Abstract

In lightweight design, the usage of different optimised materials is widespread. The interfaces between two different materials are prone to damage and, therefore, the Structural Health Monitoring (SHM) of these areas is of interest. A new method for the damage evaluation of joints is developed and validated. The released mechanical energy (RME) during static loading of a metal–composite lap shear joint is considered as a damage assessment parameter and is set into relation to the detected Acoustic Emission (AE) energy. Eleven specimens with identical geometry but different surface treatments are used to form a statistical database for the method, i.e. to calculate the energy ratio and the fluctuation range, and the twelfth specimen is used for the validation of the method. The energy ratio varies significantly, but, considering the fluctuation analysis, the RME with a known range can be predicted on the basis of the AE signal. The whole process is repeated twelve times to validate the methodology. This method can be applied to different geometries and load cases without sophisticated modelling of the damage behaviour. However, load–displacement curves of the pristine joint need to be known, and the monitored joints need to be damage-tolerant and must show similar damage behaviour.

## 1. Introduction

Reductions in weight and manufacturing costs and the extension of maintenance intervals are crucial requirements for lightweight structures [1,2]. Composite materials and material compounds are common ways to reduce weight and utilise the full capacity of materials [3,4]. Therefore, efficient joining of different materials with different material properties is necessary [5,6]. However, the interfaces between different materials are critical areas for damage initiation [7,8]. Consequently, the automatic monitoring of interfaces for damage detection and evaluation is of great interest. For this research, a new Structural Diagnosis methodology based on Acoustic Emissions (AEs) to assess damages in lap shear specimens is introduced.

In the literature, joints that use more than one joining method are called hybrid joints [9]. The structural enhancement of hybrid joints is motivated by different objectives. The delay of initial damage, slower propagation of damages, increased ultimate strength, higher absorbed energy (work-to-failure), improved damage tolerance, and stable adhesive fracture growth are a few of the reported improvements [7,10]. Therefore, the joint geometry of single-lap shear specimens (according to ASTM D5868 [11]) has been optimised to reduce stiffness discontinuities and enable adherend interlocking of protruding pins. Both arrangements (global topology optimisation and interlocking features) aim to achieve an increased failure load and advanced damage tolerance. The adherends of this research are made of the aviation industry-certified titanium alloy Ti6Al4V and carbon fibre-reinforced polymer (CFRP). In previous publications, it has been shown that the total failure load and damage tolerance under static loading are significantly increased compared to less sophisticated geometries [12,13].

However, these specimens typically fail in the interface between the two adherends and show sufficient residual strength and post-damage lifetime to allow their potential usage for aviation applications in conjunction with damage monitoring strategies. Different sensor technologies (e.g. piezoelectric transducers) and different techniques (e.g. AE) can be used for damage monitoring [1]. The choice of sensor technology and technique is dependent on many factors, for instance environmental conditions, substrate materials, dominant failure modes and size. Several Structural Health Monitoring (SHM) methods have been developed for damage monitoring and quality assessment of hybrid joints. Electrical resistance [14,15], guided waves [16], and AE measurements [17,18] are a few of the recently published approaches. In particular, advantages of AE monitoring, such as high sensitivity, real-time capability, total specimen volume sensitivity, localisation of damage regions [19], and its passive nature [20], have been documented for many years. Also, alternative Inverse Analysis identification strategies, such as Artificial Neural Networks, can be conducted on the basis of AE measurements [21]. And the correlation of AEs with different damages (e.g. impact damage, corrosion formation and crack/damage propagation [1,22]) enables its application for structural testing and surveillance, process monitoring, and material characterisation [23]. However, there are also challenges using this technique. AE monitoring does not provide an instant assessment of the extent of damage [2]. Rather, it necessitates the continuous observation of the structure over an extended period, commencing from a validated health state (for instance, at a maintenance phase). Also, noise and changing conditions (e.g. geometry, sample size, sensor distance) have a negative impact on the measurement quality [2,24].

AEs can be differentiated into primary and secondary emissions [2]. For the damage monitoring purposes in this research, primary emissions (sources internal to material, e.g. microstructural mechanisms like fracture [2]) are relevant. During damage initiation and propagation, elastically stored strain energy is released [20,25]. The released energy can, therefore, be used for damage quantification (e.g. applied in the energy release rate concept [26]) and is considered as the damage metric in this research. Plastic deformation, the formation of new surfaces, and other dissipative processes (e.g. thermal energy) consume most of the energy, and only a small fraction of this energy is converted to a transient elastic waveform called AE [20,27]. Utilising the piezoelectric effect, these strain waves can be recorded either by a commercially available AE sensor or directly by a lead zirconate titanate (PZT) element, which is often a component of a commercial AE sensor. Such PZT elements are small, lightweight, inexpensive, and additionally, have a better performance in the high-frequency region compared to commercial AE sensors [28]. In the context of a potential SHM application area, PZT elements are used for this research. During static loading of comparable advanced joints, AE events measured with PZT elements have been reported [12]. The classical approach for AE analysis is the consideration of parameters, so-called features, of the AE signal [29]. Depending on the SHM level (i.e. detection, localisation, assessment, or consequence [30]), damage mode (e.g. cohesive/adhesive failure [31], disbond, or (micro-)cracks [24]), and load collective (e.g. static and/or fatigue loading [32,33]), different features may be appropriate and correlate with damage parameters [34] (e.g. different released AE energy for cohesive/adhesive failure [31]). From a physical perspective, the AE energy is the most direct analysis approach [29], probably the most informative [32], and one of the most commonly used parameters for damage assessment [34]. Furthermore, the energy has a low dependence on the preset amplitude threshold and frequency filter values [35]. In the literature, different AE energy definitions and measurement approaches are presented (MARSE energy, absolute/true energy derived from the voltage signal [35,36,37] or a measurement device [38]). Moreover, data acquisition systems (DASs) at the current state of the art are not limited to the recording of AE parameters, but enable the storage of the whole waveform [39,40] and extended postprocessing [40] to reduce errors (e.g. calculation of root mean square (RMS) to calculate the true energy [34]). Nevertheless, AE has limitations regarding sensitivity and temporal resolution. In particular, too low sampling rates may alter the resulting waveform. According to [41], most of the energy for composite failure lies within a frequency range of 1 kHz and 1 MHz. Taking the Nyquist–Shannon sampling theorem [42] into account, sensors are typically operated with at least twice the upper limit of the frequency range. However, PZT elements and their bonding to mechanical structures also have a frequency-dependent transfer function, analysed, e.g. in [28], which need to be considered for detailed analysis.

In [43], a comprehensive review focusing on damage diagnostics and prognostics based on AEs is provided. Regarding diagnostics, the publication refers specifically to damage initiation detection, damage type identification and damage localisation of damages, whereas damage quantification plays a secondary role. For this purpose, other researchers have investigated the relationship between (cumulative) AE energy and damage propagation for different materials and applications (e.g. concrete and rocks [44], and CFRP composites [32,34]) for many years [45]. Specifically, the correlation between AE energy and mechanical strain or fracture energy was analysed [27,44,46,47]. In this context, the Sentry function as the logarithm of stored strain energy to cumulative AE energy [43] was introduced. All these authors found a correlation between AE and fracture energy, but often, big scatters between the energies were noted and only qualitative but not quantitative relations were stated [46,47]. Furthermore, most of these studies focused on the stored strain energy, or the fracture energy after total failure, and thus, did not consider the released mechanical energy during the loading process. However, researchers found a proportionality factor between accumulated AE and calculated micro-mechanical energy for coated CFRPs [27]. Although, this method is very dependent on assumed material parameters and measurement errors, and only works for geometries covered by analytical models (linear elastic fracture mechanics) [27]. A very recent publication focuses on the correlation between cumulative AE energy and the stiffness degradation as a damage propagation indicator, using finite element analysis and a predictive machine learning model [48]. Though, the experiments are conducted solely for a classical single-lap shear geometry. In sum, a method delivering a quantitative correlation between AE and released energy for geometrically advanced specimens and validated with real-world experiments is lacking.

In the present research, a new methodology for damage assessment of advanced joint geometries based on AE energy is introduced. To overcome the structural and damage complexity of such advanced joints, a robust approach that considers significant damage events rather than focusing on the detection of the smallest possible damage propagation is implemented. The method is validated with twelve tensile-tested lap shear joints. The released strain energy (considered as a feature for the inverse structural integrity of the joint) and the accumulated AE energy are calculated and their relationship is characterised. A deviation analysis is used to assess the error between predicted (based on AE measurements) and measured released energy. Utilising a probability density function, the released energy can be predicted based on the AE energy.

## 2. Materials and Methods

Prior to the introduction of the developed methodology, the specimen geometry, and the test rig and measurement methods are explained in detail.

### 2.1. Specimen Geometry

**Figure 1:**
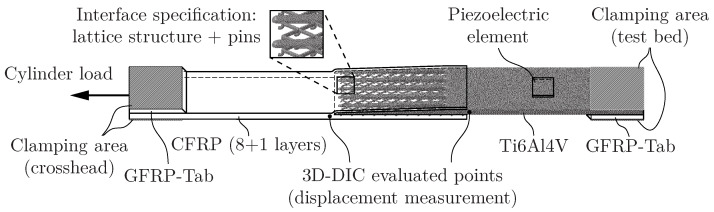
Schematic of the tested specimens.

The specimen geometry has been optimised following a biomimetic approach to reduce stiffness discontinuities and, consequently, stress concentrations on the boundaries of the overlap area (see Figure 1). On the lower surface of the tapering root structure, a lattice structure with pins on its intersections is attached to facilitate interlocking of the adherends. Enabling this complex geometry in one production stage, the metal adherend is additively manufactured with a 3D printer by selective laser melting. An EOS EOSINT^®^ M 280 laser powder bed fusion machine with the following process parameters and commercially available Ti6Al4V Grade 5 powder are used. The powder is processed by a 400 W Nd:YAG laser with a laser power of 280 W, a scan speed of 1200 mm/s, a hatch distance of 140 μm, and a layer thickness of 30 μm. Regarding the metal adherend, a few different processes are tested to alter material properties and prevent corrosion, e.g. heat treatment, sandblasting, and different coatings (hydrophilic, hydrophobic, plasma-enhanced). However, these processes and variations are beyond the scope of this work, and therefore, are not evaluated in detail. The CFRP adherend consists of a laminate made of nine layers of 2/2 twill weave fabrics (prepreg system: Delta-Tech^®^ GG200T-DT01CN-42). The laminate is cured in an autoclave together with the inlaid metal adherend. Thus, the matrix material of the prepreg is used as an adhesive and no additional adhesive is needed. Afterwards, glass fibre-reinforced polymer (GFRP) tabs are adhered with 3M^®^ Scotch-Weld^®^ DP 490 to ensure in-plane loading of the adhesive layer (see Figure 1). In total, 12 specimens with identical geometry but different metal adherend treatments are tested.

### 2.2. Experimental Setup

The specimens are tested quasi-statically on a hydraulic test rig following an in-cylinder displacement (ICD) trajectory of 0.5 mm/min. They are clamped with MTS^®^ 647 Hydraulic Wedge Grips and sandpaper is inlaid between the grips and the specimen. The tension test is conducted with a Zwick/Roell^®^ hydraulic 25 kN-cylinder. The ICD and the cylinder load are measured with a sample rate of 100 Hz.

For Structural Diagnosis purposes, a Vallen AMSY-6^®^ DAS is used for AE measurements. A 10×10×0.5 mm piezoelectric element (material: PIC^®^151; manufacturer: PI Ceramic, Lederhose, Germany) is adhesively bonded with LOCTITE^®^ EA 9466^TM^ to the surface of the titanium adherend (see Figure 1). The sensor is positioned in a manner that facilitates accessibility (e.g. for contact with the wires) while preventing an impact on the damage process or damage to the sensor itself during the clamping or loading of the specimen. Utilising the piezoelectric effect, surface strains are converted into a voltage signal, which is downsized by an in-house-developed voltage divider to avoid amplitudes beyond the saturation voltage of the measurement device. The signal is measured using continuous-mode data acquisition and is postprocessed with MATLAB^®^ R2020b. Therefore, a signal-based or quantitative AE technique [40] is applied. The parameter values chosen for the evaluation of the voltage signal are listed in Table 1. The frequency response of a PZT element is dependent on the element size, the sensing mode, and the thickness of the host structure [28], and therefore, no universal sensor frequency range is given. In anticipation of the subsequent postprocessing and in consideration of previously published research by the authors, the sampling rate is set to 5 MHz.

**Figure 2:**
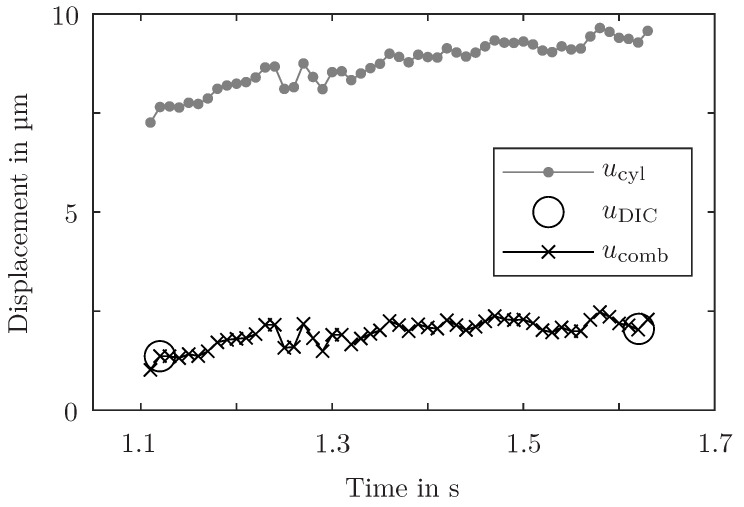
Combination of ICD measurements $u_{\mathrm{cyl}}$ and DIC displacement measurements $u_{\mathrm{DIC}}$ into combined displacement $u_{\mathrm{comb}}$. The trends are plotted paradigmatically for an arbitrary specimen and time interval.

Additionally, one lateral surface in the overlap area is coated with a primer and a speckle pattern to improve the contrast and enable Digital Image Correlation (DIC) measurements. Two 5 MP digital cameras take pictures of this surface with a sample rate of 2 Hz which are evaluated by the DIC system Correlated Solutions VIC-3D 8. For the evaluation of approximately 4000 measurement points on average, the subset size is set to 25 px, the step size to 6 px, and the average magnification is 35 px/mm. Two inspect points on both sides of the overlap region are evaluated (see Figure 1). The measurements are used to determine the actual vertical displacement of the overlap area, whereas the ICD measurements depict the accumulated elongation of the specimen, the clamping arrangement, and the cylinder rod. Furthermore, the influence of rotations of the specimen is not considered during the ICD measurement. But the sample rate of the ICD measurement is 50 times higher than the sample rate of the DIC cameras. For this reason, both measurement methods, ICD and DIC, are combined (see Figure 2). The ICD measurement is scaled with the vertical displacement measurements of the DIC to combine the benefits of both methods.

### 2.3. Evaluation Method and Procedure

**Figure 3:**
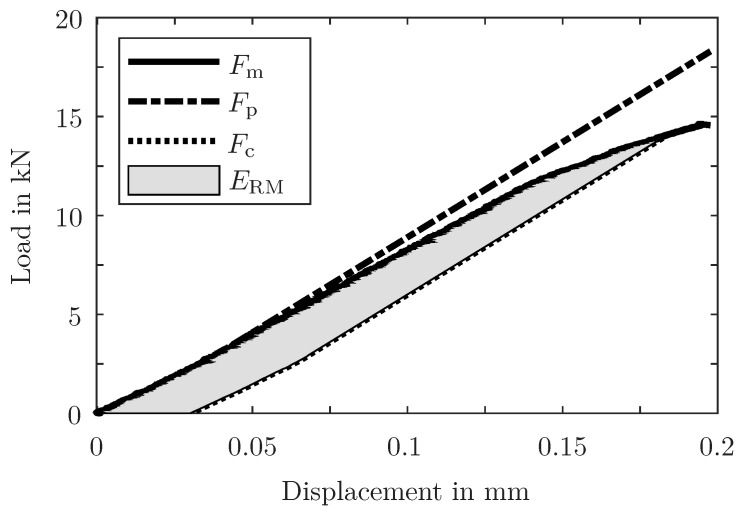
Determination of the RME $E_{\mathrm{RM}}$ (at an arbitrary displacement *u*) of one specimen from the load–displacement curve. The measured load $F_{m}$ and the conservative load $F_{c}$ (parallel translation of the pristine specimen load $F_{p}$) are subtracted and integrated. The pristine specimen load $F_{p}$ is identical to the measured load $F_{m}$ until its maximum stiffness (i.e. gradient) is reached and then extrapolated with this gradient.

For the assessment of the health state of the joint, the released mechanical energy (RME) is evaluated. This energy is calculated based on the area below the load–displacement curve of the specimen (see Figure 3):

(1) $$\begin{array}{cc} \Delta E_{\mathrm{RM}}\left(t_{0},T\right) & =E_{\mathrm{RM}}\left(t_{0}+T\right)-E_{\mathrm{RM}}\left(t_{0}\right)\\ & =\int_{u(0)}^{u(t_{0}+T)}\left(F_{m}\left(u\right)-F_{c}\left(u,t_{0}+T\right)\right)du-\int_{u(0)}^{u(t_{0})}\left(F_{m}\left(u\right)-F_{c}\left(u,t_{0}\right)\right)du. \end{array}$$

The energy of an electric signal is calculated by integrating the electric resistance multiplied by the squared voltage signal over time. In general, the resistance is assumed to be constant, and therefore, scales the energy as a constant factor. As the absolute AE energy in Joule is merely an intermediate result for the introduced method (to predict the RME), this constant factor can be disregarded:

(2) $$\Delta E_{\mathrm{AE}}\left(t_{0},T\right)=\int_{t_{0}}^{t_{0}+T}V^{2}\left(t\right)dt={\left(\sqrt{\frac{1}{T}\int_{t_{0}}^{t_{0}+T}V^{2}\left(t\right)dt}\right)}^{2}T={\left(V_{\mathrm{RMS}}\left(t_{0},T\right)\right)}^{2}T.$$

The AE events measured during the load increase are synchronised with the test rig measurements (load and ICD). However, not only damage initiation or damage propagation may cause AE events. But, the objective of this research is to achieve robust predictions based on a macroscopic view. For this reason and for a reduction in data, only significant AE events are considered. On average, 20% of the detected AE events contribute to 99% of the total AE energy; the remaining 80% of the detected events are considered insignificant. The AE filtering parameters, frequency centroid $f_{c}$ and peak amplitude $\hat{V}$, are selected in order to achieve this differentiation (see Table 1).

**Figure 4:**
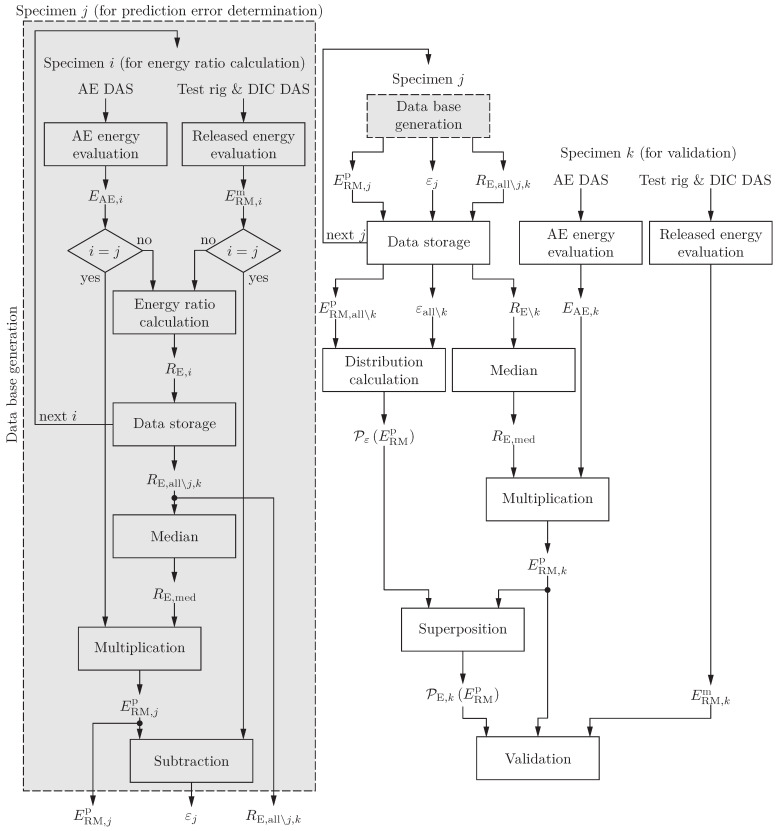
Flowchart for the prediction database generation, the fluctuation evaluation and the validation of the method. The database is generated with eleven specimens (i.e. j∈{1,…,11}) and the validation is made with the twelfth specimen (i.e. k∈{12}). $E_{\mathrm{RM}}^{m}$ represents the measured RME, whereas $E_{\mathrm{RM}}^{p}$ stands for the predicted RME (based on AE measurements). $R_{E,\mathrm{all}\setminus j,k}$ depicts the energy ratios of all events of all specimens without specimen *j* and specimen *k*. $P_{\varepsilon }\left(E_{\mathrm{RM}}^{p}\right)$ is the probability distribution of the errors of all but *k* specimens $\varepsilon_{\mathrm{all}\setminus k}$ and $P_{E,k}\left(E_{\mathrm{RM}}^{p}\right)$ is the predicted RME probability distribution of specimen *k*.

For robust results, the energy ratio

(3) $$R_{E}\left(t_{0},T\right)=\frac{\Delta E_{\mathrm{RM}}\left(t_{0},T\right)}{\Delta E_{\mathrm{AE}}\left(t_{0},T\right)},$$

is calculated for each AE event of 10 specimens and the median of all energy ratios is formed (see Figure 4). The 11th specimen is used to compare the actual RME (from load and combined displacement measurements) and the predicted RME (based on the AE energy and the median energy ratio). The error between these energies is used to estimate the fluctuation range of the method. For this reason, the error (11 error trends for 11 specimens) related to the predicted RME is used to make probability distributions for a certain energy level, i.e. the error values are grouped with the predicted RME. The methodology is validated with the measurement results of the 12th specimen (see Figure 4). According to the standard for testing the lap shear adhesion of fibre-reinforced plastic (FRP) bonding (ASTM D5868 [11]), at least five lap shear samples are necessary, and also for the fracture toughness determination of FRPs (ASTM D5528 [49]), five specimens per test condition need to be tested. Given that the tested specimens are not a standard geometry, the number of samples is doubled, and it is assumed that the stochastic failure process is covered.

## 3. Results

The developed methodology is tested on basis of measurement results of twelve specimens. First, the mechanical measurements and the comparability of these measurements are shown. Then, the AE measurements and, especially, the preprocessing of the results are analysed. Afterwards, the energy evaluations of both measurement domains (structural/test rig and SHM/AE) are compared, and differences and challenges are appraised. Based on these findings, the energy ratio between RME and detected AE energy is calculated and evaluated. In the end, the energy ratio is taken to predict the RME and show the reliability of the developed methodology for the damage assessment of hybrid joints.

### 3.1. Structural Specimen Behaviour

**Figure 5:**
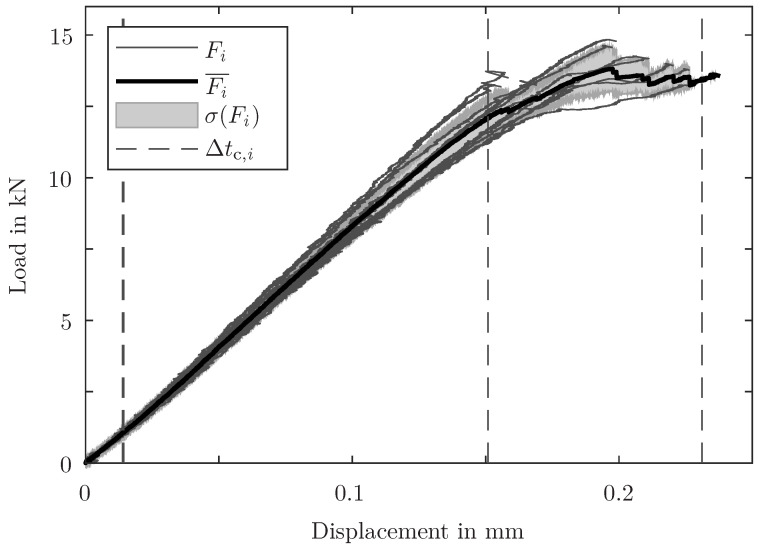
Load $F_{i}$ over combined displacement $u_{\mathrm{comb}}$ of all tested specimens. The descriptive statistics of the loads, mean load $\bar{F_{i}}$ and standard deviation $\sigma (F_{i})$, indicate the comparability of the tested specimens. The CTPs $\Delta t_{c,i}$ (considered for further evaluations) are shown for the specimens with the smallest and the biggest displacements.

The ICDs are controlled, but the actual vertical displacement of the joining region deviates. However, the specimens show similar load–displacement curves and total failure loads (see Figure 5). This indicates a reproducible manufacturing quality and a predictable damage behaviour, even though different metal adherend processing steps (e.g. coating variations) were applied and the displacement at total failure varies significantly. Specimens of the same material with the same geometry are comparable in terms of mechanical response on static loading. The restriction of the evaluation time to the considered time period (CTP) in between the dashed lines (see Figure 5) is made to ensure consistent framework conditions. In the beginning, the load is too low for relevant damage events, whereas in the end, the final fracture process is accompanied by a superposition of many different effects. Therefore, AE sources in both time intervals are assumed to be not directly related to major damage events and, for this reason, are not considered in this method. This time limitation is also used in the following figures.

### 3.2. Acoustic Emission Measurements

**Figure 6:**
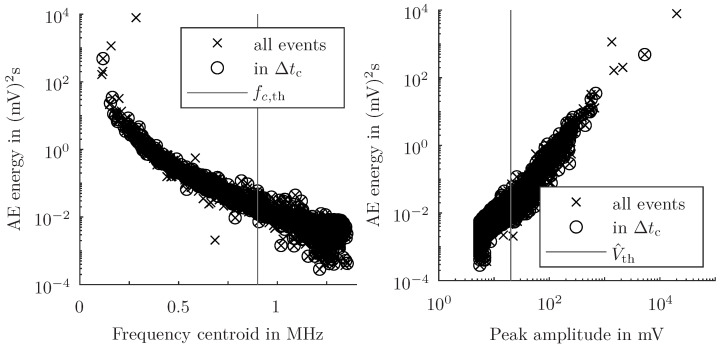
(**Left**) Correlation between AE energy $E_{\mathrm{AE}}$ and frequency centroid $f_{c}$ (incl. the chosen frequency centroid threshold $f_{c,\mathrm{th}}$). (**Right**) Correlation between AE energy $E_{\mathrm{AE}}$ and peak amplitude $\hat{V}$ (incl. the chosen peak amplitude threshold ${\hat{V}}_{\mathrm{th}}$) of all detected AE events, and the detected events in the CTP $\Delta t_{c}$ for one specimen.

The continuously measured voltage signal generated by the piezoelectric element on the surface is evaluated as mentioned in the previous section. Besides the energy, two characteristic parameters, the frequency centroid $f_{c}$ and the peak amplitude $\hat{V}$, of each identified AE event are determined. In Figure 6, the AE energy is plotted against these two parameters, considering the CTP. The AE events in the CTP show a relationship and have distinct cut-off values. The frequency centroid is constrained by the sampling rate and the peak amplitude is limited by the threshold value $V_{\mathrm{th}}$ chosen for the AE event identification. As mentioned in the previous sections, the introduced method is directed at robust results based on major damage events, and therefore, does not aim for as small as possible damage events. For filtering purposes, either the frequency centroid threshold $f_{c,\mathrm{th}}$ is defined to $9\times 10^{5}\;\mathrm{Hz}$ or the peak amplitude threshold ${\hat{V}}_{\mathrm{th}}$ is set to 20 mV. As a result, on average, the number of detected AE events of 3.752 is reduced to 3.177 events during the CTP. Moreover, the filtering lowers the number of events to 538 ($f_{c}<9\times 10^{5}\;\mathrm{Hz}$) or 614 ($\hat{V}>20\;mV$) on average per specimen. Both parameters can be used to filter the raw data and suppress minor energy events. Depending on the chosen thresholds, there is no significant difference between both filtering parameters and both filter the same events.

### 3.3. Released and Acoustic Emission Energy

**Figure 7:**
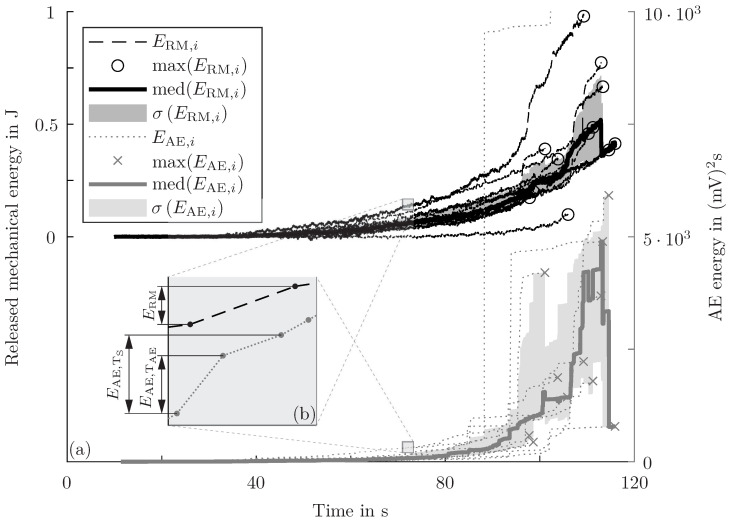
(**a**) Comparison of RME $E_{\mathrm{RM},i}$, and detected and $f_{c}$-filtered AE energy $E_{\mathrm{AE},i}$ of all tested specimens during the CTP. The descriptive statistics of the trends (median trend $\mathrm{med}\left(E_{i}\right)$ and standard deviation $\sigma \left(E_{i}\right)$) and the energy at the end of the CTP $\max\left(E_{i}\right)$ are included. (**b**) Schematic detail to illustrate the two methods: single AE event evaluation $E_{{\mathrm{AE},T}_{\mathrm{AE}}}$ and sample point evaluation $E_{{\mathrm{AE},T}_{S}}$.

The energy of the detected and $f_{c}$-filtered AE events and the RME, based on the combination of test rig and DIC measurements, of all tested specimens during the CTP is shown in Figure 7a. Both energies increase over time, but the (relative) energy increments are not equal. Also, the standard deviation of the energies rises. Therefore, the energies need to be evaluated in detail. Only a fraction of the RME is converted into AE energy and only a fraction of this AE energy can be detected below the area of the sensor. Consequently, the energy detected is smaller than the total RME. Different units are used for the different energies. The electric AE energy can be scaled by a constant factor to obtain the strain energy stored below the sensor (see the explanation for Equation (2) in the previous section). This step is not undertaken because for the prediction of the RME based on AE measurements, this intermediate result (i.e. strain energy below the sensor) is not necessary. The markers in Figure 7a depict the total failure of single specimens and justify jumps and drops in the mean trends of the energies. The correlation of strain changes, as an indicator for damage propagation, and AE events has been shown for comparable specimens in a previous publication [12], and hence is not repeated here for the sake of brevity.

**Figure 8:**
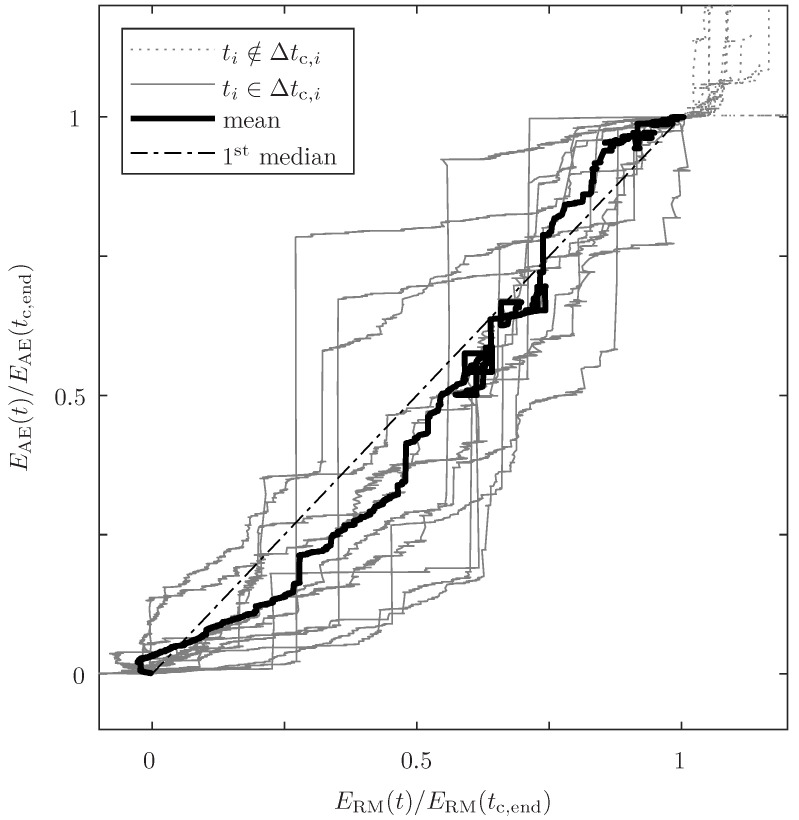
Relative AE energy (accumulated AE energy/total accumulated AE energy) over relative RME (accumulated RME/total accumulated RME).

To illustrate the relationship between RME and detected AE energy, the relative accumulated energy (ratio of accumulated energy to total energy in the end of the CTP) of both domains is shown in Figure 8. Although the mean relation is close to the ideal correlation (e.g. on average, 80% of the mechanical energy is released simultaneously to the detection of 80% of the AE energy, following the first median in Figure 8), for some specimens, the correlation deviates greatly. In extreme cases, 78% of the AE energy is detected, while only 27% of the RME is measured. As mentioned in the introduction, in other studies, big scatters of the damage process-related energies were also obtained. Due to the fact that more than double the number of samples are tested compared to the relevant standards mentioned, it is assumed that the full spread is captured. In brief, there is no constant relationship between RME and detected AE energy and, therefore, the ratio between both energies needs to be analysed.

The energy ratio of RME to detected AE energy is calculated for each event, and two different evaluation methods are compared. First, each identified, filtered AE event and the RME increment between the sample points before and after the event are used for the energy ratio (“single AE event evaluation” using $E_{{\mathrm{AE},T}_{\mathrm{AE}}}$; see Figure 7b). In particular, a 3 ms lasting AE event is set in relation to the RME during a 10 ms sample time (100 Hz sample frequency). Second, the accumulated, filtered AE energy in between two sample points is set in relation to the increment RME between the same sample points (“sample point evaluation” using $E_{{\mathrm{AE},T}_{S}}$; see Figure 7b). Specifically, the (accumulated) AE energy and the RME of the same 10 ms time window are put into a relation. The advantage of the single AE event evaluation is that there is no grouping of different damage events to calculate the energy ratio. However, the RME sample rate is not large enough for an assignment of single damage events, and consequently, the energies of two different time periods are compared. The sample point evaluation enables a congruence of the considered time intervals. The median energy ratios of both approaches are used for the prediction of the RME to compare both methods at the end of the chapter. The calculated energy ratios over time show huge variations and no distinctive trend over time. Furthermore, the median energy ratio based on the whole measurement time and the CTP does not change significantly (for both methods). The same is found for the two implemented filtering methods ($f_{c}$- vs. $\hat{V}$-threshold).

### 3.4. Energy Prediction

**Figure 9:**
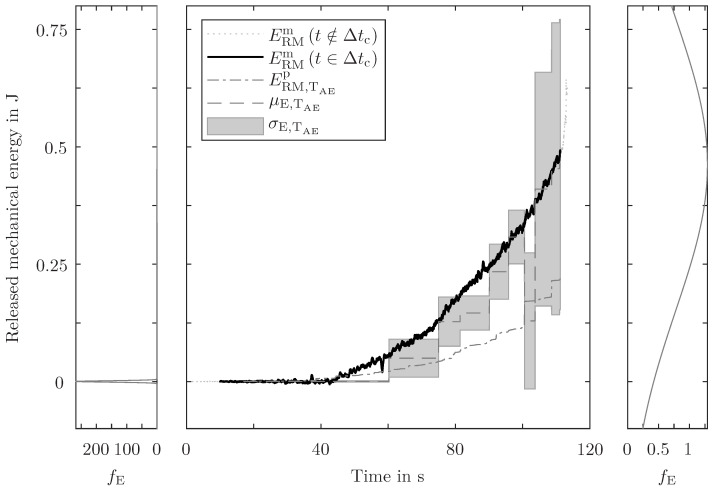
Comparison of measured ($E_{\mathrm{RM}}^{m}$) and predicted ($E_{\mathrm{RM}}^{p}$) RME for one arbitrarily chosen specimen, based on the single AE event evaluation ($T_{\mathrm{AE}}$). From the fluctuation evaluation $P_{E}\left(E_{\mathrm{RM}}^{p}\right)$, the expected value $\mu_{{E,T}_{\mathrm{AE}}}$ and the standard deviation $\sigma_{{E,T}_{\mathrm{AE}}}$ are depicted in the centre plot. The measured RME outside the CTP ($t\notin \Delta t_{c}$) is illustrated as a dotted line. In the left and right diagrams, the probability density functions of the predicted energy $f_{E}$ at the very beginning and at the very end of the CTP are displayed.

**Figure 10:**
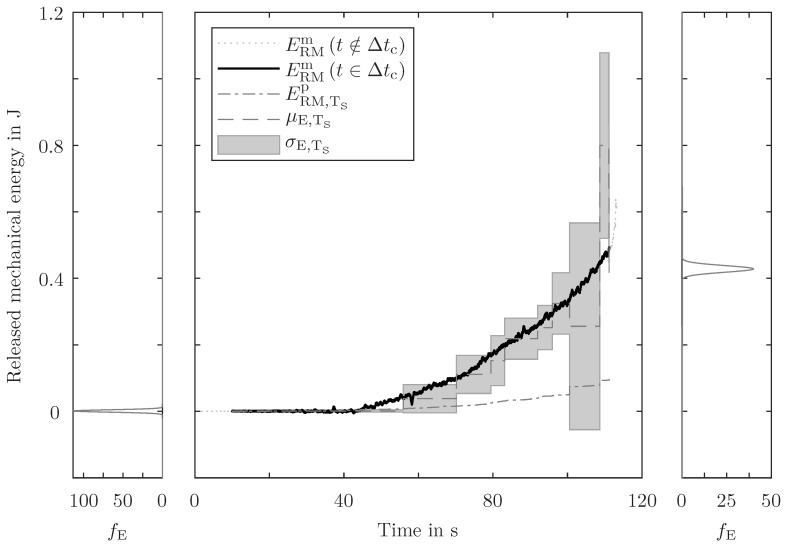
Comparison of measured ($E_{\mathrm{RM}}^{m}$) and predicted ($E_{\mathrm{RM}}^{p}$) RME for one arbitrarily chosen specimen, based on the sample point evaluation ($T_{S}$). From the fluctuation evaluation $P_{E}\left(E_{\mathrm{RM}}^{p}\right)$, the expected value $\mu_{{E,T}_{S}}$ and the standard deviation $\sigma_{{E,T}_{S}}$ are depicted in the centre plot. The measured RME outside the CTP ($t\notin \Delta t_{c}$) is illustrated as a dotted line. In the left and right diagrams, the probability density functions of the predicted energy $f_{E}$ at the very beginning and at the very end of the CTP are displayed.

In Figure 9 and Figure 10, for one randomly selected specimen, the measured RME is compared with the predicted RME. The predictions use the $f_{c}$-filtered AE energy and both mentioned methods are conducted (single AE event evaluation in Figure 9 and sample point evaluation in Figure 10).

Often, there is only one single AE event between two sample points. But there are sample time periods with more than one detected AE event in them. Therefore, the energy ratio of the single event evaluation is bigger than the energy ratio of the accumulated energy evaluation. The detected AE energy is multiplied by the determined energy ratio. The grouping into different sample time periods does not have an influence on the accumulated energy and, therefore, the prediction based on the single event evaluation is always higher than the prediction based on the sample point evaluation (bigger energy ratio and same detected—but differently grouped—AE energy result in higher prediction). The predictions of both methods do not always match the measured RME. The fluctuation evaluation of both methods for the start and the end of the prediction is shown as probability density functions on the left and right sides of the prediction graphs. Despite the different (median) predictions, the expected value of both methods is close to the measured RME. The single AE event evaluation method has a higher resolution, but the evaluated time intervals are not coherent (e.g. 3 ms for AE and 10 ms for RME measurements). Whereas, the sample point evaluation has a lower resolution but coherent time intervals. The procedure is conducted twelve times, i.e. for each evaluation, another specimen is used for validation, and therefore is suppressed for database generation. The whole CTP needs to be examined to assess the prediction quality of this method; however, to obtain an easily interpretable metric, only the end of the CTP is appraised. Predictions with more than 3 σ deviation between expected and measured RME and more than a 50% relative error are considered poor predictions. For the single AE event evaluation, four out of twelve, and for the sample point evaluation, five out of twelve predictions are poor. Overall, and with consideration of the fluctuation evaluation, neither method is preferred and both deliver good energy prediction results.

## 4. Discussion

The previous section shows the applicability of the presented methodology for damage assessment purposes. However, there are certain limitations for this method and the performance quality varies. Therefore, in this section, the results of the previous section are discussed and put into context with the existing literature. Also, further steps and potential improvements are presented.

### 4.1. Application Limitations

For this study, all specimens are manufactured in the same way and are tested under the same conditions (e.g. same cylinder trajectory, same clamping situation). However, the metal adherends are processed differently (heat treatment, sandblasting, coating), which highlights the robustness of this method. Identically treated specimens would deliver even better results, i.e. predictions with smaller fluctuation ranges. The requirements for this method are the same conditions; although, the impact of variations regarding the mentioned parameters are not evaluated in detail due to a limited number of available specimens.

The main challenges of this method are the identification of the actual RME and how much of this energy can be released prior to the total failure of the joint. Both aspects are crucial for the assessment objective and the applicability of this method. For this joint geometry and these materials, the pristine load–displacement curve is appraised, but for other specifications, the calculation of the pristine energy might be more challenging. Additionally, the discretisation and quality of the measurements (e.g. noise, distance between sensor and AE source) have a significant impact on the reliability of this method.

There is a huge variation in calculated energy ratios between RME and AE energy. And, the fluctuation range of both energies (see Figure 7) and the missing linear relationship between the accumulated energies (see Figure 8) are challenging preconditions for a robust prediction. In particular, the calculation of the RME is prone to errors. Furthermore, the dominant damage mode, the AE transition behaviour, the damping, and the energy propagation may be dependent on the damage source location and the stress state. However, there is no distinct trend of energy ratios over time. Other filter parameters (e.g. peak frequency) are also tested, but the best results are attained with the two mentioned parameters ($f_{c}$ or $\hat{V}$). In sum, it is beneficial to have big AE events during damage propagation facilitated by a damage-tolerant design. But, unpublished results show the applicability of this method for even less damage-tolerant designs which are more closely related to classical single-lap shear specimens.

### 4.2. Context in the Literature

The damage progression in composite materials is complex, and therefore, no universal relationship between cumulative AE energy and crack length has been introduced yet [34]. Only a fraction of the RME is converted into AEs, and therefore, complementary monitoring of other energies which also correlate with damage formation, e.g. released thermal energy [50], may support a more complete understanding of the damage process. However, dissipative energies other than AEs are not considered in this research. A major advantage of this method is that the crack growth law need not be known in detail, but the damage behaviour only needs to be repeatable. Additionally, the difficult comparison and extrapolation of results of different researchers [34] are not necessary. Instead, the method is trained for the individual application (e.g. hybrid joint) without previous knowledge. The big variation in the energy ratio (equivalent to proportionality constant α in [27]) has been observed and is caused by error-prone reference values (delamination area in [27]). The developed methodology of this research delivers a solution for this challenge and a step towards practical applicability in the form of a fluctuation evaluation.

### 4.3. Outlook

The application of this method for different geometries and load cases (e.g. out-of-plane loading, dynamic loading) is a feasible next step. Also, a more sophisticated filtering approach to distinguish between primary and secondary emissions enhances the practicality. Furthermore, the combination of two evaluation methods (i.e. the superposition of the probability densities in Figure 9 and Figure 10) and the usage of identical specimens (with identical coatings) may bring even better prediction results. For the generalisation and extrapolation of this methodology to other geometries, the calculation of the actual strain energy below the sensor can be a step towards decoupling the database generation from real-world application.

## 5. Conclusions

To gain an improved applicability of hybrid joints as part of a lightweight approach, a new Structural Diagnosis methodology is introduced. The health state of the joint is considered to be inversely proportional to the RME. This energy cannot be measured easily, especially not during operation. Therefore, the measured AE energy is evaluated and put in proportion to the RME. With the aid of a deviation analysis, the RME can be predicted based on the AE measurements and the damage state of a joint can be assessed. Depending on the method, for eight or seven out of twelve advanced lap shear specimens, the predictions deliver good results, i.e. the predicted and measured energies at the end of the CTP differ by less than 3 σ or by less than 50% relative error. The method is individually applicable to other geometries and use cases. Without more information, it is possible to form a database for the structural assessment of joints with the same geometry and load case. However, monitored joints need to be damage-tolerant and have similar damage behaviour.

The main results of this research are summed up below:

- A methodology for the assessment of the structural integrity of advanced geometries, in particular hybrid joints, based on AE energy is introduced.
- It is not necessary to have detailed prior knowledge of the damage modes that may occur; rather, the introduced method employs a robust, macroscopic approach.
- The RME, the AE energy, and the relation between both energies are calculated and vary significantly. As a solution for this challenge, a deviation analysis is implemented.
- Utilising a signal-based approach, the AE energy can be used for the prediction of the RME of advanced hybrid joints with a complex geometry.
- For data-efficient postprocessing of the AE signal, several filtering approaches are analysed. A maximum frequency centroid and a minimum peak amplitude of the evaluated AE events are proper filtering parameters.

## Figures and Tables

**Table 1:** AE Evaluation Parameters.

AE event identification						
	Sample rate	Duration		Rise time	Threshold	
	*f*	$t_{\mathrm{period}}$		$t_{\mathrm{rise}}$	$V_{\mathrm{th}}$	
	5 MHz	3 ms		0.1 ms	5 mV	
AE event filtering				Time evaluation restriction		
Frequency centroid	Peak amplitude			Start drop time		End drop time
$f_{c}$	$\hat{V}$			$\Delta t_{\mathrm{start}}$		$\Delta t_{\mathrm{end}}$
0.9 MHz	20 mV			10 s		2 s

## Data Availability

Data will be made available on request.

## Abbreviations

The following abbreviations are used in this manuscript:

AE	Acoustic Emission
AM	additively manufactured
CFRP	carbon fibre-reinforced polymer
CTP	considered time period
DAS	data acquisition system
DIC	Digital Image Correlation
FRP	fibre reinforced plastic
GFRP	glass fibre-reinforced polymer
ICD	in-cylinder displacement
NDE	Non-destructive Evaluation
PZT	lead zirconate titanate
RME	released mechanical energy
RMS	root mean square
SHM	Structural Health Monitoring
